# The Effect of Natural Organic Matter on Mercury Methylation by *Desulfobulbus propionicus* 1pr3

**DOI:** 10.3389/fmicb.2015.01389

**Published:** 2015-12-18

**Authors:** John W. Moreau, Caitlin M. Gionfriddo, David P. Krabbenhoft, Jacob M. Ogorek, John F. DeWild, George R. Aiken, Eric E. Roden

**Affiliations:** ^1^School of Earth Sciences, University of MelbourneMelbourne, VIC, Australia; ^2^United States Geological SurveyMiddleton, WI, USA; ^3^United States Geological SurveyBoulder, CO, USA; ^4^Department of Geology and Geophysics, University of Wisconsin-MadisonMadison, WI, USA

**Keywords:** mercury methylation, methylmercury, sulfate-reducing bacteria, natural organic matter, mercury isotopes

## Abstract

Methylation of tracer and ambient mercury (^200^Hg and ^202^Hg, respectively) equilibrated with four different natural organic matter (NOM) isolates was investigated *in vivo* using the Hg-methylating sulfate-reducing bacterium *Desulfobulbus propionicus* 1pr3. *Desulfobulbus* cultures grown fermentatively with environmentally representative concentrations of dissolved NOM isolates, Hg[II], and HS^−^ were assayed for absolute methylmercury (MeHg) concentration and conversion of Hg(II) to MeHg relative to total unfiltered Hg(II). Results showed the ^200^Hg tracer was methylated more efficiently in the presence of hydrophobic NOM isolates than in the presence of transphilic NOM, or in the absence of NOM. Different NOM isolates were associated with variable methylation efficiencies for either the ^202^Hg tracer or ambient ^200^Hg. One hydrophobic NOM, F1 HpoA derived from dissolved organic matter from the Florida Everglades, was equilibrated for different times with Hg tracer, which resulted in different methylation rates. A 5 day equilibration with F1 HpoA resulted in more MeHg production than either the 4 h or 30 day equilibration periods, suggesting a time dependence for NOM-enhanced Hg bioavailability for methylation.

## Introduction

The organometallic neurotoxin methylmercury (MeHg) inhibits human fetal and adult neurological development and cellular functionality (e.g., Marsh et al., 1980; Mergler et al., 2007; Newland et al., 2008; Ceccatelli et al., 2010). Human exposure to MeHg primarily occurs via consumption of contaminated fish (Skerfving, 1988; Sunderland, 2007) in which MeHg has bioaccumulated through heterotrophic lacustrine or marine food webs (Monteiro and Furness, 1997; Fitzgerald et al., 2007; Merritt and Amirbahman, 2009). Much of the consumed MeHg originates as anthropogenic inorganic mercuric mercury, Hg(II) (e.g., Schuster et al., 2002), that is atmospherically deposited into aquatic ecosystems with anoxic bottom sediments. Environmental mercury (Hg) methylation results primarily from the anaerobic transformation of Hg(II) by sulfate-reducing bacteria (SRB) (e.g., Jensen and Jernelöv, 1969; Compeau and Bartha, 1985; Goulet et al., 2007), although some iron-reducing bacteria (IRB), methanogens, and members of the *Firmicutes* phylum may also be important environmental methylators (Fleming et al., 2006; Kerin et al., 2006; Hamelin et al., 2011; Gilmour et al., 2013; Yu et al., 2013). Recent studies have identified and characterized a functional gene cluster (*hgcAB*) for Hg methylation in microorganisms (Parks et al., 2013; Poulain and Barkay, 2013). The role of this gene in biogeochemical Hg cycling, as well as its evolutionary origin and ecological distribution, require further study. Whatever the methylating mechanism(s) and biogeochemical pathway(s), microbial Hg methylation has been an active area of research for over 30 years because of its complexity and central importance to the global Hg cycle (Fitzgerald et al., 2007; Selin, 2009; Sonke et al., 2013).

Significant advances in our knowledge of the microbial Hg methylation process have been made over the last two decades (e.g., Gilmour and Henry, 1991; Choi and Bartha, 1993; Choi et al., 1994a,b; Ekstrom et al., 2003; Schaefer and Morel, 2009; Brown et al., 2011a,b; Gilmour et al., 2011; Parks et al., 2013). Still, major gaps persist in our understanding of the geochemical forms of inorganic Hg available for biomethylation (Benoit et al., 2003; Jonsson et al., 2012; Hsu-Kim et al., 2013), the biochemical pathways involved (Landner, 1971; Choi et al., 1994b; Poulain and Barkay, 2013), and the mechanism for uptake of Hg by methylating microbes (Benoit et al., 1999, 2001a; Schaefer and Morel, 2009; Bridou et al., 2011; Schaefer et al., 2011, 2014; Pedrero et al., 2012). Numerous studies have established that the bioavailability of aqueous Hg(II) strongly depends on its speciation, which is in turn influenced primarily by the presence and composition of natural organic matter (NOM) and sulfide (Benoit et al., 1999, 2001c; Ravichandran et al., 1999; Drexel et al., 2002; Ravichandran, 2004; Miller et al., 2007; Slowey, 2010; Jonsson et al., 2012; Zhang et al., 2012; Hsu-Kim et al., 2013). However, other studies have highlighted the potential for enhanced mercury methylation due to Hg release from iron-ox(yhyrox)ide colloids, or via a yet undetermined link to the metabolism of IRB (Fleming et al., 2006). Recent work (Gilmour et al., 2013) illustrates the environmental diversity of known anaerobic Hg-methylating microbes, including methanogens, acetogens, and obligate syntrophs. Other recent studies propose that anaerobes may not play an important role in mercury methylation in the open ocean (Malcolm et al., 2010), or that aerobes may methylate mercury as well (Larose et al., 2010). These findings carry new implications for environmental factors influencing mercury bioavailability, although interactions with NOM will most likely play an important role across all ecosystems.

Interactions with NOM have been observed to produce variable effects on Hg methylation, including inhibition (Barkay et al., 1997; Watras et al., 1998; Hammerschmidt and Fitzgerald, 2004; Hammerschmidt et al., 2008), promotion (Furutani and Rudd, 1980; Driscoll et al., 1994; Watras et al., 1998; Cai et al., 1999; Lambertsson and Nilsson, 2006; Schartup et al., 2012), and no effect (Hurley et al., 1998). Indeed, recent work has shown Hg can even undergo redox transformations when interacting with NOM reactive sites (Gu et al., 2011; Zheng et al., 2012; Chakraborty et al., 2015) that may be important for constraining Hg bioavailability for methylation. Previous studies have examined the relationships between Hg(II) methylation and other environmental reactants such as aqueous (bi)sulfide (Benoit et al., 2001a,b; Jay et al., 2002; Jeremiason et al., 2006; Sunderland et al., 2006; Zhang et al., 2012), sulfate loading (Drevnick et al., 2007), and the role of microbial community structure in methylation (Pak and Bartha, 1998a,b; King et al., 2000, 2001; Macalady et al., 2000). In addition, the effect of variable NOM composition on Hg(II) bioavailability has recently been examined under controlled experimental conditions with certain representative microorganisms and/or in the presence of aqueous (bi)sulfide (Merritt and Amirbahman, 2009; Graham et al., 2012, 2013; Pham et al., 2014). In this study, we expand on previous work to investigate the effect of four well-characterized NOM isolates, each having a different functional group composition (Table 1), on Hg bioavailability for methylation by a sulfate-reducing bacterium, *Desulfobulbus propionicus* strain 1pr3 (DSM 2032 and ATCC 33891).

**Table 1:** **Compositional data for natural organic matter isolates (compiled from Waples et al., 2005; Boyer et al., 2008)**.

**NOM isolate**	**wt%**						**MW**	**Aliphatic I**	**Aliphatic II**	**Acetal**	**Aromatic**	**Carboxyl**	**Ketone**	**Reduced S**	**SUVA_280_L**	**Source**
	**C**	**H**	**O**	**N**	**S**	**Ash**	**Da**	**0–62 ppm**	**62–90 ppm**	**90–110 ppm**	**110–160 ppm**	**160–190 ppm**	**190–230 ppm**	**mol% total S**	**(mg C)^−1^cm^−1^**	
Suwannee River Humic Acid (SRHA)	53.4	3.9	40.9	1.1	0.68	4.13	1399	21.3	7.3	6.6	35.1	20.7	9.0	18.3	0.0547	Black water draining Okeefenokee Swamp near Fargo, Georgia Vegetation: Southern Floodplain Forest (Quercus, Nyassa, Taxodium)
Williams Lake Hydrophobic Acid (WL HpoA)	52.7	5.2	36.6	1.7	0.72	2.98	772.0	50.0	15.0	5.8	13.8	13.9	1.5	nd	0.0132	Seepage lake, north-central Minnesota. Organics dominated by autochthonous sources (algae, bacteria, emergent vegetation)
F1 Hydrophobic Acid (F1 HpoA)	52.2	4.64	39.9	1.53	1.73	9.37	1031	33.1	8.9	2.3	25.4	23.1	7.2	28.7	0.0309	Eutrophied marshland in Water Conservation Area 2A, northern Everglades. Vegetation dominated by Cattails (26°21′35” N; 80°22′14”W)
F1 Transphilic Acid (F1 TpiA)	47.7	4.1	44	2.5	1.6	9.37	832	43.5	20.6	6.4	13.1	14.6	1.8	nd^*^	nd^*^	Same as F1 HpoA

* *not determined*.

## Materials and methods

### Culture conditions

**Figure 1:**
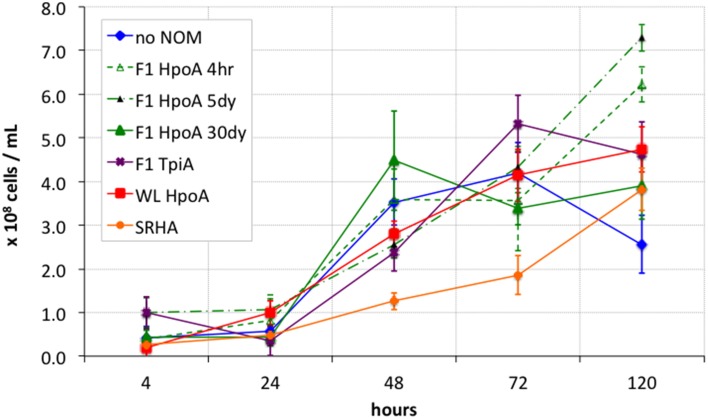
**Growth curves for bacterial Hg methylation experiment**. Cell densities were calculated from optical microscopy-calibrated OD600 measurements of inoculated cultures. OD600 measurements from respective cell-free controls were subtracted from those of inoculated cultures to account for absorption effects from NOM only. Killed cell control showed < 10^7^ cells mL^−1^ throughout entire experiment. Error bars represent one standard error on the mean of 2-3 replicates.

*In vivo* methylation assays were conducted using the SRB *D. propionicus* 1pr3 (DSM 2032) grown fermentatively. *D. propionicus* 1pr3 can methylate Hg at a high rate relative to other SRB and non-SRB methylators (King et al., 2000; Benoit et al., 2001a), and can be maintained in culture using either respiratory or fermentative media. The use of fermentative media (with pyruvate as both the carbon and electron source) allows for control of ambient bisulfide (HS^−^) to environmentally realistic concentrations (~10 μM), which could otherwise accumulate under sulfate respiration to influence Hg speciation separately (Benoit et al., 2001b). Other studies have shown higher MeHg yields for SRB when grown fermentatively instead of by sulfate respiration (Goñi-Urriza et al., 2015; Perrot et al., 2015). For this study, *D. propionicus* 1pr3 cells were grown in a fermentative growth medium with no sulfate (after Benoit et al., 2001a), and transferred at late exponential growth phase between 3 and 5 times before inoculation into the experiment. A small amount of yeast extract (1% v/v) was required to sustain fermentative growth across the pre-experimental transfers. The Ti-nitrilotriacetic acid reductant of Benoit et al. (2001a) was omitted to avoid an observed precipitation of TiO_2_ floc that could act as an adsorbent for aqueous Hg(II), dissolved NOM, or *D. propionicus* cells, or interfere with spectrophotometric assays for HS^−^ concentration and cell density. Cell densities for *D. propionicus* 1pr3 were measured throughout the experiment using optical (cell) density at 600 nm wavelength (“OD600”) absorption measurements on a Bio-Rad SmartSpec spectrophotometer calibrated to *D. propionicus* 1pr3 cell morphology by direct cell counting on a Neubauer counting chamber slide. All cultures and controls were incubated in 100 mL of medium sparged with N_2_ and crimp-sealed over butyl rubber septa prior to autoclaving. All culturing experiments were performed in triplicate, while cell-free and killed cell negative control treatments were prepared in experimental triplicate and duplicate, respectively. Cell-free controls consisted of uninoculated medium containing an NOM isolate, while the killed-cell control consisted of cells autoclaved prior to their transfer into experimental medium with NOM isolate “F1 HpoA 30dy.” Cell-free controls displayed neither growth nor Hg methylation during the experiment. The OD600 measurements for this negative control type have been subtracted from those of the respective live cultures (i.e., experimental conditions *with* cells) to remove the effect of NOM absorption, and to facilitate inspection of cell growth curves (Figure 1). Anaerobic culturing bottles were 120 mL borosilicate serum bottles (Bellco) that were acid washed with 6N HCl at 50°C overnight before triple rinsing with milli-Q water and double-bagging in trace metal clean plastic zip-type bags until use.

### NOM characteristics and handling

Environmental NOM isolates (Table 1) were obtained using methods described by Aiken et al. (1992), and include: Suwannee River Humic Acid (SRHA; Suwannee River, Georgia), Williams Lake Hydrophobic Acid (WL HpoA; White Oak, Minnesota), F1 Hydrophobic Acid (F1 HpoA; Florida Everglades), and F1 Transphilic Acid (F1 TpiA; Florida Everglades). For discussion of the methods by which the physical and chemical properties of each NOM isolate were characterized, see Waples et al. (2005) and Boyer et al. (2008). The isolates were selected on the basis of variability in sulfur content and degree of aromaticity, and specifically the nature of various functional groups (e.g., carboxyl, thiols) that could interact with aqueous Hg(II) (see Table 1 for details).

NOM isolates were weighed out in a freeze-dried form and dissolved into sterile anoxic (sparged with N_2_) milli-Q water. Each NOM isolate was injected into autoclaved and cooled experimental media via syringe filtration (0.22 μm pore size cellulose acetate filters) to a final concentration of ~40 mg L^−1^. Following NOM isolate addition, cultures were amended with 5–10 μM of filter-sterilized aqueous HS^−^ (quantified using the methylene blue method of Cline (1969) on a Hach® DR890 portable spectrophotometer) to simulate environmental ambient HS^−^ concentrations.

### Hg methylation assays

Approximately 24 h after amendment with NOM isolate and sulfide, aqueous ^200^Hg(II) was added as a tracer (as ^200^HgCl_2_) to each culture or control bottle to a final concentration of 100 ± 10 ng L^−1^. In addition, the F1 HpoA isolate was used to assay the effect of different ^200^Hg(II) pre-equilibration times (4 h, 5 days or 30 days in the presence of ~5 μM aqueous bisulfide) on MeHg production; these experimental conditions are referenced as “F1 HpoA 4hr,” “F1 HpoA 5dy,” and “F1 HpoA 30dy,” respectively. All other NOM isolates were equilibrated with ^200^Hg(II) in the dark for 30 days to simulate long exposure periods and/or slow flow rates through anoxic lacustrine or estuarine sediments. After equilibration, pH values were checked in bottles designated as negative (cell-free) controls. With the exception of the “no NOM” cell-free and killed-cell controls (pH 6.9–7.0), all NOM isolate-amended controls showed pH values of 7.1–7.2 (Table 2). Aqueous sulfide concentrations were measured ~24 h after addition to culturing bottles, at the beginning of the experiment prior to inoculation, and again at ~48 h incubation time. Roughly 5 μM HS^−^ was initially added to each culture or control, but in some bottles additional HS^−^ was required at the beginning of the experiment (prior to inoculation) to maintain the cultures at an ambient HS^−^ concentration of 5–10 μM. Over the course of the experiment, observed concentrations of aqueous HS^−^ increased by at most a factor of two in most cultures, and a factor of three in a few (see Table 3), possibly resulting from the small amount of yeast extract that was transferred from pre-experimental growth culture vessels (Benoit et al., 2001a).

**Table 2:** pH values (±0.01) at t_0_^*^ for medium in duplicate negative controls (−1, −2).

	**−1**	**−2**
no NOM	6.9	7.0
SRHA	7.2	7.2
WL HpoA	7.2	7.2
F1 HpoA 4hr	7.2	7.2
F1 HpoA 5dy	7.1	7.1
F1 HpoA 30dy	7.2	7.2
F1 TpiA	7.2	7.2
Killed cells		

* *Prior to inoculation*.

**Table 3:** **Maximum ${\text{HS}}_{\left(\text{aq}\right)}^{-}$ concentrations (mg/L) in triplicate live cultures (+1, +2, +3) and duplicate negative controls (−1, −2)**.

	**+1**	**+2**	**+3**	**−1**	**−2**
*t* = 0 h^*^					
no NOM	0.16	0.16	0.16	nd	nd
SRHA	0.16	0.16	0.24	nd	nd
WL HpoA	0.16	0.32	0.16	nd	nd
F1 HpoA 4hr	0.16	0.16	0.16	nd	nd
F1 HpoA 5dy	0.16	0.32	0.24	nd	nd
F1 HpoA 30dy	0.16	0.16	0.16	nd	nd
F1 TpiA	0.16	0.16	0.16	nd	nd
Killed cells	0.40	0.24	n/a	n/a	n/a
*t* = 48 **h**					
no NOM	0.48	0.40	0.40	0.24	0.16
SRHA	0.32	0.32	0.32	0.24	0.24
WL HpoA	0.40	0.32	0.32	0.24	0.16
F1 HpoA 4hr	0.40	0.32	0.48	0.32	0.32
F1 HpoA 5dy	0.32	0.32	0.32	0.08	0.24
F1 HpoA 30dy	0.48	0.32	0.32	0.24	0.16
F1 TpiA	0.40	0.40	0.32	0.16	0.16
Killed cells	0.32	0.32	n/a	n/a	n/a

* *Prior to inoculation; n/a, not applicable; nd, not determined*.

**Figure 2:**
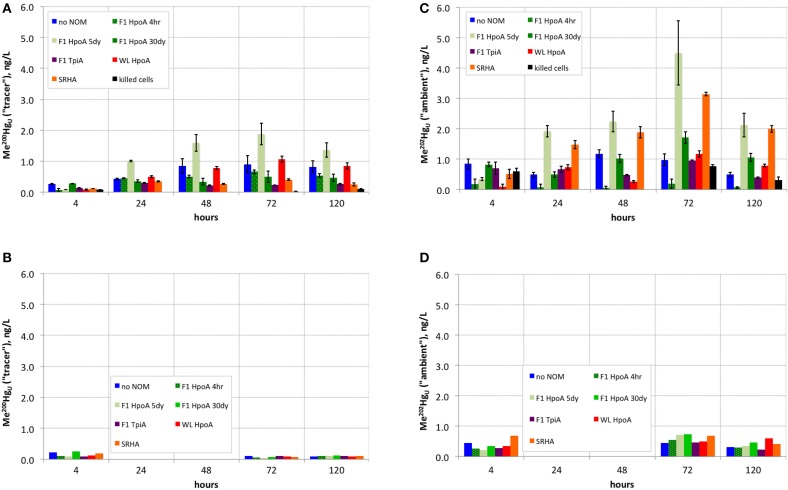
**(A)** Absolute concentration of methylmercury produced from ^200^Hg(II) tracer over time in experimental live cultures. **(B)** Negative controls (single replicate) for **(A)**. **(C)** Absolute concentration of methylmercury produced from ambient ^202^Hg(II) over time in experimental live cultures. **(D)** Negative controls (single replicate) for **(C)**.

**Figure 3:**
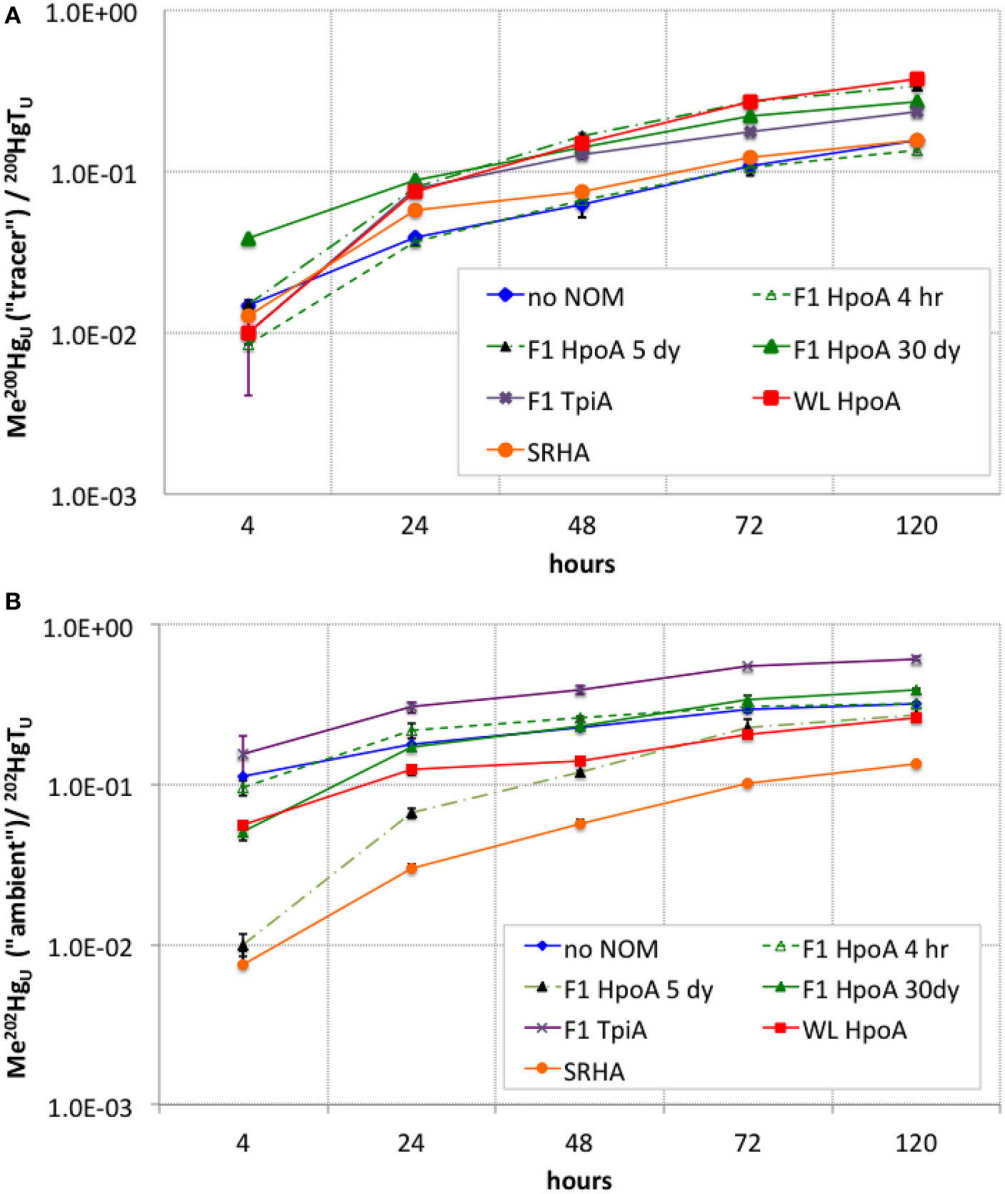
**(A)** Cumulative conversion of ^200^Hg(II) tracer to Me^200^Hg, normalized to total unfiltered ^200^Hg, for each NOM isolate culturing condition. **(B)** Cumulative conversion of ambient ^202^Hg(II) to Me^202^Hg, normalized to total unfiltered ^202^Hg, for each NOM isolate culturing condition. The subscript “_U_” indicates the measurements represent unfiltered MeHg concentrations at each sampling timepoint. Error bars represent one standard error on the mean of 2–3 replicates.

**Figure 4:**
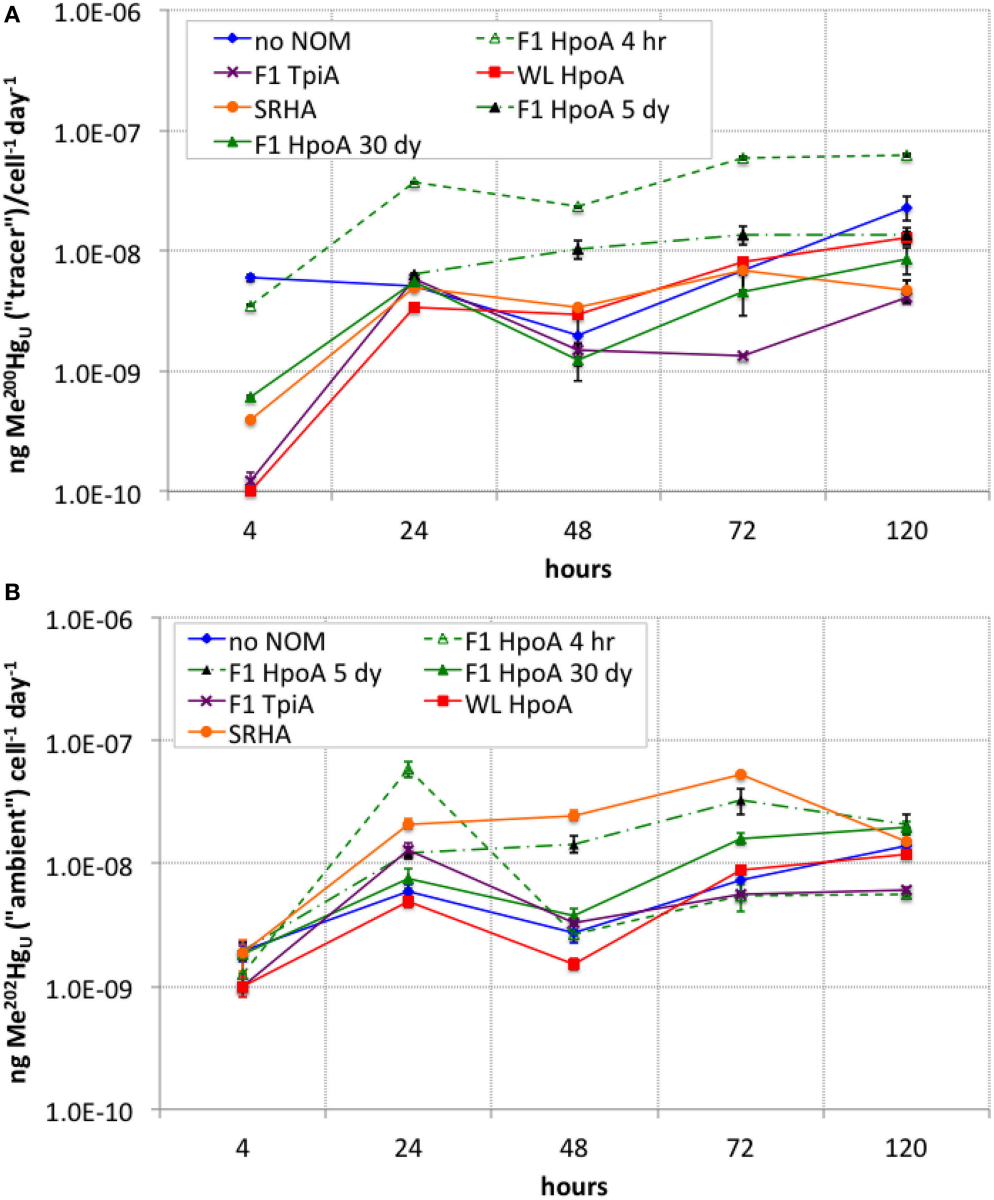
**(A)** Cell density-normalized rates of Me^200^Hg production for each NOM isolate culturing condition. **(B)** Cell density-normalized rates of methylation of ambient Me^202^Hg for each NOM isolate culturing condition. The subscript “_U_” indicates the measurements represent unfiltered MeHg concentrations at each sampling timepoint. Error bars represent one standard error on the mean of 2–3 replicates.

Cultures were inoculated with 1 mL of *D. propionicus* 1pr3 cells (10^8^–10^9^ cells mL^−1^) transferred at late exponential growth phase. Sampling for concentration of bacterial cells (OD600), total unfiltered Hg(II), and unfiltered MeHg analyses was performed at 4, 24, 48, 72 and 120 h after inoculation for live growth culture bottles, and at 4, 72, and 120 h post-inoculation for negative controls and killed-cell incubations, with 5 and 10 mL of each culture or control taken for unfiltered MeHg and total Hg analyses, respectively. Mercury methylation potential was assessed in three ways. We measured and plotted the unfiltered (“_U_”) concentrations (in ng L^−1^) of Me^200^Hg_U_ (i.e., enriched ^200^Hg isotope tracer) and Me^202^Hg_U_ (i.e., “ambient” ^202^Hg naturally present in each NOM isolate) over time to assess the absolute Hg methylation potential (Figure 2). We also measured unfiltered concentrations of total Hg(II), ^200^HgT_U_ and ^202^HgT_U_, and plotted the ratios of Me^200^Hg_U_/^200^HgT_U_ and Me^202^Hg_U_/^202^HgT_U_, to assess the cumulative proportion of methylation of each mercury isotope over time (Figure 3). Finally, methylated mercury isotope concentrations were normalized to optically calibrated cell densities for *D. propionicus* 1pr3, in order to approximate cell-specific methylation rates at each sampling timepoint (Figure 4).

### Hg analyses

All Hg and MeHg analyses were performed at the USGS Mercury Research Laboratory in Middleton, Wisconsin (http://wi.water.usgs.gov/mercury-lab/). Total Hg analysis was performed using U.S. EPA Method 1631. Aqueous samples were pre-treated with 1–2 % (v/v) 0.2N bromine monochloride (BrCl) to solubilize and oxidize all forms of Hg to reactive mercuric mercury (Hg[II]). Samples were placed in an oven at 50°C for a minimum of 12 h to accelerate the oxidation reaction. Oxidation was considered complete if excess BrCl was present (faint yellow color) after 12 h. If necessary, additional BrCl was added and allowed to react for another 12 h. Just prior to analysis, a small amount of hydroxylamine hydrochloride (NH_2_OH–HCl) was added to each sample until any residual color from the BrCl disappeared. Approximately 10 min after BrCl reduction, about 125 mL of sample was poured into a bubbling flask and 0.5 mL of stannous chloride (SnCl_2_) was added to reduce the Hg(II) to gaseous elemental Hg (Hg[0]). The Hg(0) was then purged from the sample with Hg-free N_2_ gas and concentrated onto a gold-coated, glass-bead trap. Finally, the gold trap was heated and the Hg(0) thermally desorbed into an argon gas stream and quantified by cold vapor atomic fluorescence spectrometry (CVAFS).

MeHg analyses were performed using a distillation and ethylation procedure (e.g., Horvat et al., 1993; DeWild et al., 2002) with addition of a precisely known amount of methylated Hg isotope (Me^199^Hg) to allow for assessment of recoveries on an individual sample basis. All MeHg in each sample was isolated by water vapor distillation under atmospheric pressure. MeHg in distillates was then derivatized using sodium tetraethylborate and pre-concentrated onto Carbotraps™. Detection and quantification was achieved after thermal desorption, isothermic GC separation, and detection by ICP-MS (Hintelmann and Evans, 1997). For these samples, 30 picograms of Me^199^Hg were added to each sample about 30 min before the distillation step to serve as a sample-specific internal standard. Because detection was performed using an ICP-MS, the Me^199^Hg was quantified independently from the Me^200^Hg tracer and ambient Me^202^Hg. Following quantification of the measured Me^199^Hg, all the other Hg isotope masses were corrected based on the internal standard recovery ratio. Individually applied recoveries allowed us to correct for widely variable recoveries commonly observed for tracer addition checks on performance, random errors associated with derivatization and detection steps, and drifts in instrument response during a run.

Despite the presence of NOM isolates in all but two experimental conditions (no NOM and killed-cell controls), and the presence of 5–10 μM aqueous sulfide in all bottles, we acknowledge that a significant portion of the total dissolved ^200^Hg may have been lost to adsorption on the glass walls of the culturing bottles (Graham et al., 2013). We speculate that the cleaning procedure to prepare the bottles for this experiment, which involved overnight washing in 6N HCl at 60°C, could have attacked the glass to produce sorption sites for aqueous Hg(II). However, the effect would be a reduction in the total amount of the ^200^Hg tracer available for methylation (i.e., the effect would be present in Figure 2). Comparison of methylation efficiency in the presence of different NOM isolates was still possible on the basis of quantifying the proportion of Hg methylated (Figure 3) and cell-specific methylation rates (Figure 4).

## Results

### Cell growth

All cultures exhibited a lag phase during the first 24 h of growth (Figure 1). Around 48 h into the experiment, most cultures (except SRHA) showed growth to a cell concentration roughly within one standard error of the other culture conditions. For the later timepoints (72 and 120 h), cells cultured with NOM isolates F1 HpoA 4hr, F1 HpoA 5dy, F1 HpoA 30dy, and WL HpoA (30 day) showed more growth than did cells cultured with SRHA or no NOM. Cells also exhibited higher growth in the presence of F1 TpiA toward the latter half of the experiment. In general, all cultures grew to lower densities than were obtained when *D. propionicus* was provided with propionate as the carbon substrate and sulfate as the terminal electron acceptor (data not shown).

### Methylation of the Hg(II) “tracer” (^200^Hg)

Cultures containing ^200^Hg(II) equilibrated with the F1 HpoA 5dy NOM isolate exhibited the most Hg(II) tracer methylation (~2 ng L^−1^ Me^200^Hg; Figure 2A). Cultures containing 30-day pre-equilibrated WL HpoA also exhibited substantial Me^200^Hg production (>1 ng L^−1^). Other cultures that exhibited notable ^200^Hg(II) methylation were the NOM-free, F1 HpoA 4hr, and F1 HpoA 30dy experimental conditions (~0.8, ~0.6, and ~0.5 ng L^−1^ Me^200^Hg, respectively). Less ^200^Hg was methylated in cultures containing F1 TpiA (~0.3 ng L^−1^ Me^200^Hg) and SRHA (~0.4 ng L^−1^ Me^200^Hg) NOM isolates. Cell-free controls and killed-cell controls contained less than ~0.25 ng L^−1^ Me^200^Hg (Figure 2B).

The highest cumulative proportion of methylation of ^200^Hg(II) was observed in cultures containing WL HpoA and F1 HpoA 5dy NOM isolates (~0.35–0.37, Figure 3A), although the proportion of ^200^Hg methylated in the presence of WL HpoA was initially much lower. F1 HpoA 30dy and F1 TpiA cultures also showed substantial cumulative proportion of methylation of ^200^Hg over the course of the experiment (0.23–0.27, Figure 3A). Lower cumulative proportion of methylation was observed in cultures containing no NOM, SRHA, and F1 HpoA 4hr (~0.12–0.15, Figure 3A). Killed-cell controls showed virtually no ^200^Hg methylation (< 0.1; data not shown).

The cell-specific rate of Hg tracer methylation (Figure 4A) was highest (~5.9 attograms ^200^Hg methylated cell^−1^ day^−1^) at the start of the experiment for the “no NOM” culture, but was higher overall during the experiment for cultures incubated with either F1 HpoA 4hr or F1 HpoA 5dy (~3.5–62 ag ^200^Hg methylated cell^−1^ day^−1^). The slowest overall cell-specific methylation rate (~1.3 ag ^200^Hg methylated cell^−1^ day^−1^) occurred in cultures incubated with F1 TpiA (Figure 4A).

### Methylation of “ambient” Hg (^202^Hg)

Interestingly, more ambient Hg (^202^Hg) than tracer Hg (^200^Hg) was methylated (i.e., Me^202^Hg produced) in the F1 HpoA 5dy NOM isolate-incubated culture and SRHA isolate-containing incubations (Figure 2C). WL HpoA and F1 HpoA 30dy cultures, as well as the “no NOM” culture, produced between 1 and 2 ng L^−1^ Me^202^Hg, but the other NOM isolate cultures exhibited less ambient methylation when compared to tracer Hg (^200^Hg) methylation rates. Less than ~1 ng L^−1^ of total Me^202^Hg was measured in killed-cell incubations (Figure 2C) and all negative controls (data not shown).

The greatest cumulative proportion of unfiltered ambient Hg (^202^Hg) methylation was observed in cultures incubated with F1 TpiA NOM across the whole of the experiment (~0.15–0.60, Figure 3B). Other NOM isolates showed variation in the cumulative percentage of ^202^Hg methylation (e.g., ~0.32–0.39 for all F1 HpoA-containing incubations).

The cell-specific rate of ambient Hg (^202^Hg) methylation was highest (~58 ag ^202^Hg methylated cell^−1^ day^−1^) near the beginning of the experiment (after ~24 h incubation period) for the culture grown in F1 HpoA 4hr. However, after ~24 h incubation, the cell-normalized methylation rate of this culture was exceeded by the SRHA and F1 HpoA 5dy cultures at rates of ~33–53 ag ^202^Hg methylated cell^−1^ day^−1^. By the final sampling time, all cultures but the F1 TpiA and F1 HpoA 4hr NOM isolate incubations exhibited cell-specific methylation rates of ~12–20 ag ^202^Hg methylated cell^−1^ day^−1^ (Figure 4B).

## Discussion

Cell growth data (Figure 1) showed that most of the cultures with the respective NOM isolates grew at similar rates, starting at ~1 × 10^8^ cells mL^−1^ density or slightly less, and increasing to between 2 × 10^8^ and 5 × 10^8^ cells mL^−1^ over 120 h. Although not clearly defining an exponential growth curve, the pattern of growth observed during the experiment was identical to that displayed during successive transfers, for which ~120 h (5 days) was sufficient to describe most or all growth to a late exponential or early stationary phase. Only *D. propionicus* 1pr3 grown in the presence of the SRHA NOM isolate grew more slowly, e.g., by a factor of 2–4 times between 48 and 72 h. This observation suggests a potentially inhibitive effect on cell growth from the SRHA NOM isolate, possibly also due to its relatively high metals content (Aiken, *personal communication*). We cannot rule out, alternatively, that some NOM isolates actually facilitated cell growth to some degree, possibly by acting as electron acceptors for pyruvate oxidation (e.g., Coates et al., 1998; Jiang and Kappler, 2008), which would result in the same apparent effect in Figure 1. The observation that cultures incubated with F1 HpoA, for example (except for the 30 dy Hg equilibration condition), seemed to be increasing still in cell density at 120 h incubation, while other cultures' cell densities approached their maxima or, in the case of the “no NOM” condition, began to decrease, supports this alternative hypothesis. More work is required in order to understand the potential effect(s) of different NOM isolates on cell growth and physiology, and the implications of such effects on Hg methylation.

Quantification of isotopically resolved non-methylated and methylated Hg(II) species (Figure 2) demonstrated the greater extent of methylation of newly added (“tracer”) ^200^Hg(II) when associated with F1 HpoA and to some extent WL HpoA, in contrast to ^200^Hg(II) equilibrated with, e.g., SRHA or F1 TpiA NOM isolates (Figures 2A, 3A). In fact, more of both the tracer (^200^Hg) and ambient (^202^Hg) mercury pools, in absolute concentration, was methylated in the presence of F1 HpoA 5dy NOM isolate than with any other NOM isolate (Figures 2A,C). These observations support the interpretation that the NOM isolate, F1 HpoA, possesses compositional and/or functional properties that promote more Hg(II) methylation overall, relative to Hg(II) methylation potential in the presence of the other NOM isolates tested. This interpretation is consistent with the observations of a previous study (Graham et al., 2013), which also found that the F1 HpoA NOM isolate promoted the highest degree of Hg(II) methylation. Those authors noted the relatively large size and high degree of aromaticity associated with the F1 HpoA dissolved NOM isolate as factors that seemed to promote Hg(II) methylation.

In contrast to the pronounced effect of F1 HpoA, however, it is worth noting that substantial Me^200^Hg was also formed when “no NOM” was used. This result was perhaps a bit surprising as previous workers (e.g., Graham et al., 2013) have observed less adsorption of aqueous Hg(II) to bottle walls in the presence of NOM, therefore postulating an overall enhancement of methylation in the presence of *any* NOM relative to the absence of any NOM. However, the absence of NOM here did not seem to have a negative impact on absolute ^200^Hg(II) methylation relative to ^200^Hg(II) methylation in the presence of all other NOM isolates (Figure 2A). Possibly some Hg(II) that would have adsorbed to bottle walls was associated with pyruvate or cell surfaces, or *D. propionicus* 1pr3 was still able to methylate adsorbed ^200^Hg(II).

Whereas, both the tracer and ambient Hg(II) pools were methylated to relatively higher levels in the presence of F1 HpoA (Figure 2), substantially less ^200^Hg(II) was methylated in the presence of the SRHA NOM isolate (Figure 2A). Overall lower cell growth in cultures incubated with SRHA (Figure 1) may also have contributed to this latter observation (Figure 2A), but we note that others have similarly observed that SRHA did not promote Hg(II) methylation relative to NOM-free controls (Biswas et al., 2011). Figure 2C reveals that more ambient Hg(II) than tracer Hg(II) was methylated in the presence of the SRHA NOM isolate, potentially explaining and reconciling these observations. This interpretation is consistent with our other observations that (a) a relatively larger ambient Hg(II) pool must be associated with the SRHA NOM isolate (Figure 3B) and (b) cell-normalized ^202^Hg methylation rates were nearly an order of magnitude higher than ^200^Hg methylation rates (Figure 4B). Apparently, *D. propionicus* 1pr3 cells grown in the presence of SRHA spent a considerable portion of the time involved in methylating Hg in producing MeHg from the ambient Hg pool associated with this NOM isolate.

Generally, we observed that more ambient ^202^Hg(II) was methylated than tracer ^200^Hg(II) by the same NOM isolate cultures (Figures 2A,C), and an even greater ratio of methylated ^202^Hg(II)/^200^Hg(II) was observed for the killed-cell control (Figure 2C). This observation supports the interpretation that substantial ambient (i.e., natural background) ^202^Hg(II) associated with the NOM isolates was bioavailable for methylation in nearly every culturing condition, possibly after early methylation of the ^200^Hg tracer (Figure 4A) and/or reflective of the lower natural abundance (subtracted from all ^200^Hg concentrations during analysis of ICP-MS data) of the *ambient* ^200^Hg(II) pool associated with each NOM isolate (Figure 3A). The exception was less ^202^Hg(II) methylated in association with the F1 HpoA 4hr NOM isolate (Figure 2C). One implication of this finding is that the bioavailability of “new” Hg (e.g., the ^200^Hg tracer) for methylation may depend not only on the size and composition of NOM in the environment, but also on the concentration and bioavailability of “aged” Hg (e.g., ambient Hg) already associated with that NOM. A natural environment likely harbors more than one “pool” of Hg bioavailable for methylation (i.e., new vs. aged Hg(II), or Hg(II) that is labile or tightly bound to different NOM functional groups), for which the bioavailability of each Hg pool depends partially on that of one or more others.

The observation that different absolute concentrations of methylated ambient Hg(II) were formed when the same NOM isolate was incubated with tracer Hg(II) and live *D. propionicus* cells for different time periods (Figure 2C) is both intriguing and a little perplexing. Examining the effect of F1 HpoA NOM isolate-Hg(II)-HS^−^ equilibration time on Hg(II) methylation also revealed that the tracer ^200^Hg was methylated most efficiently in cultures pre-equilibrated for 5 days prior to inoculation. This observation hints at a possible time-dependency for the bioavailability of newly added Hg(II), possibly related to changes in the form of Hg associated with the NOM isolate and/or the conformation of the isolate over time. Although the cumulative proportion of ^200^Hg(II) converted to Me^200^Hg in association with the F1 HpoA 30dy condition appears relatively higher than with other NOM isolates or with F1 HpoA 4hr or 5dy (Figure 3A), inspection of absolute Me^200^Hg concentrations (Figure 2A) and cell-normalized rates of ^200^Hg methylation (Figure 4A) reveal the explanation for this apparent contradiction to be the relative lower bioavailability of the aged (i.e., 30 dy) tracer Hg pool (added ^200^Hg). A related problem involves reconciliation of the observation that F1 HpoA 5dy cultures exhibited the greatest amount of ambient Hg methylation (Figure 2A), although the “ambient” Hg fraction has clearly been partitioned to this NOM isolate for a longer time. We speculate that possibly as more (new) ^200^Hg was transformed into Me^200^Hg, a stochastic increase in the efficiency of methylation for F1 HpoA ambient Hg(II) was also achieved, or alternatively that the conformation of the F1 HpoA NOM (5 day) isolate was altered during equilibration and/or incubation in such a way as to facilitate ambient Hg(II) methylation. In any case, we cannot fully explain why naturally aged Hg(II) was associated with a muted response in MeHg production relative to methylation of the ^200^Hg isotope tracer, but we speculate that the aging process itself may have somehow impacted the bioavailability of Hg(II). Our hypothesis is perhaps consistent with recent findings that aging of nanoparticulate HgS leads to reduced methylation potential (Zhang et al., 2012, 2014; Pham et al., 2014). More recently, Manceau et al. (2015) showed the abiotic conversion of Hg(II) bound to thiol groups in NOM to nanoparticulate metacinnabar over a week-to-month time scale under environmentally relevant conditions. This process may, in part, explain our result of less methylation of the (“ambient”) ^202^Hg naturally present in the NOM isolates, when compared to methylation of the ^200^Hg tracer. Indeed, if nanoparticulate β-HgS (metacinnabar) was present in some NOM isolates used in this experiment, then the aggregation state of this phase (an outcome of natural aging of nanoparticles or colloids) should also, in theory, exert influence over its bioavailability for methylation.

The cumulative proportion of Hg(II) that was methylated was plotted for both tracer and ambient Hg(II) in order to assess the overall efficiency of conversion of Hg(II) to MeHg during our experiment (Figure 3). The shape of these curves suggests a tapering of methylation rate or efficiency over the course of the experiment (i.e., after 24–48 h), or possibly the effect of quasi-simultaneous Hg demethylation. In a parallel experiment using a Me^201^Hg tracer, however, we did not observe any demethylation by *D. propionicus* 1pr3 (data not shown), although other strains of *D. propionicus* are known to demethylate MeHg (Rodríquez-Gonzáles et al., 2009), and further work is needed to confirm our result for strain 1pr3. On the other hand, we did observe a similar tapering of Hg(II) methylation rates for both tracer and ambient Hg(II) over time (Figure 4), suggesting that the cumulative percentage of Hg methylated approached an assymptotic limit in most or all NOM isolate cultures. Interestingly, the conversion of F1 TpiA-associated ^202^Hg(II) to Me^202^Hg was greater than that of any other NOM isolate, despite constituting only a moderate amount of absolute Me^202^Hg production (Figures 2C, 3B), indicative of a more efficient conversion of less total ambient Hg(II) to MeHg. It is perhaps interesting to speculate whether changes in the form of NOM-bound ^200^Hg(II) may have occurred over the course of the experiment to affect the bioavailability of the ^200^Hg tracer for methylation.

**Figure 5:**
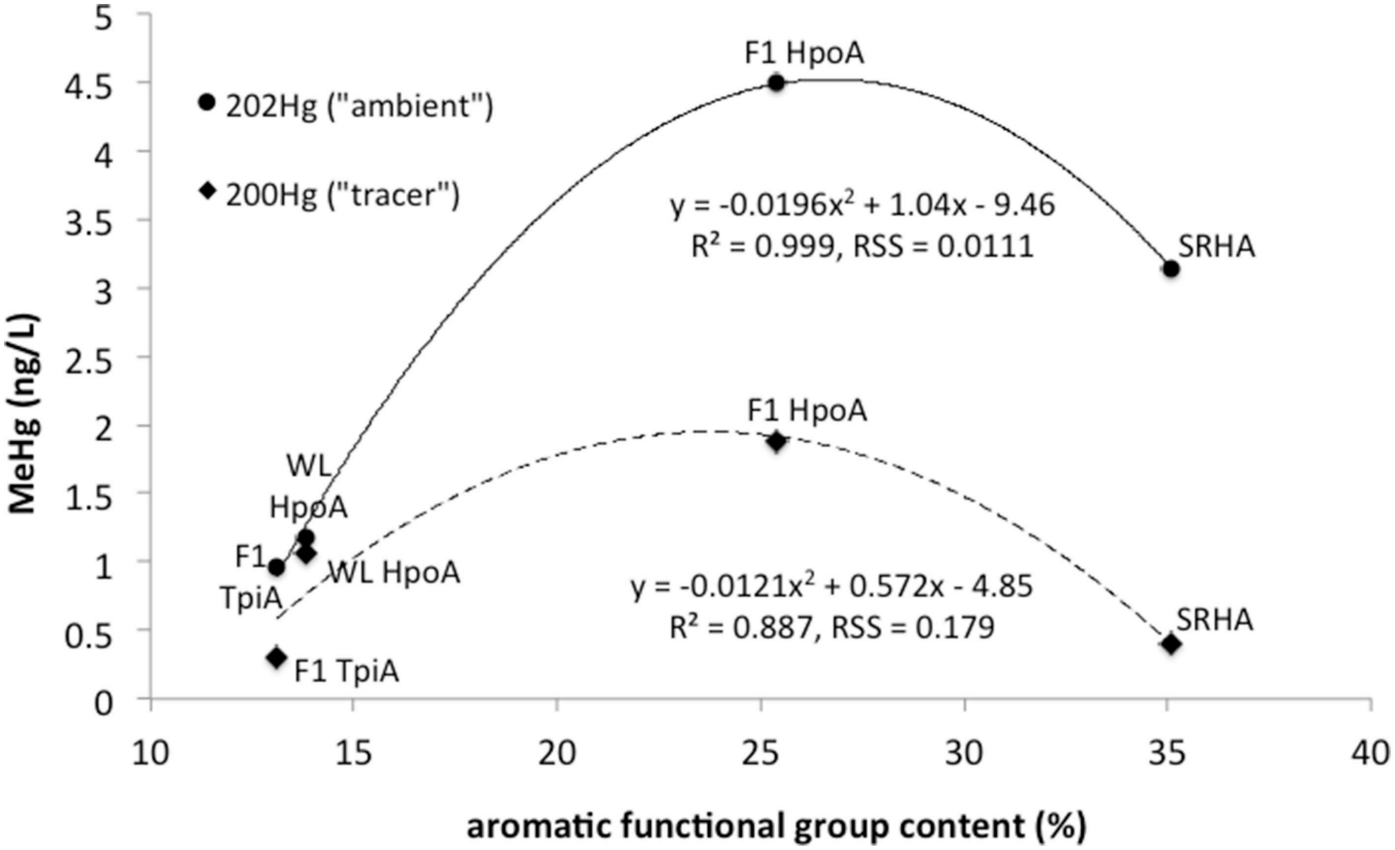
**Maximum mean absolute concentrations of methylmercury (MeHg) produced from either ^200^Hg tracer or ambient ^202^Hg vs. aromatic functional group content (%) for each NOM isolate: F1 HpoA, F1 TpiA, WL HpoA, and SRHA**. Quadratic regressions are plotted showing *R*^2^ values and Residual Sum of Squares (RSS) for goodness of fit estimation.

As shown in Table 1 and Figure 5, the NOM isolates selected for this experiment increase in their percentage of aromatic functional groups (a key contributor to hydrophobicity) as follows: F1 TpiA, WL HpoA, F1 HpoA, and SRHA. We note that the Hg:DOM ratio used in our study, ~30 pmol Hg (mg NOM)^−1^, is about two orders of magnitude lower than that used by Gerbig et al. (2011) to determine mercury speciation under environmentally relevant levels of aqueous NOM and sulfide. Interestingly, Graham et al. (2013) found contrasting results for methylation by *Desulfovibrio desulfuricans* ND132 in the presence of F1 TpiA and WL HpoA, and reported SUVA_254_ values for these NOM isolates of 2.93 ± 0.04 and 1.95 ± 0.02, respectively (although the reported sulfur contents are roughly equivalent to those presented in Table 1 here). Another similar study by these authors (Graham et al., 2012) also found contrasting results for the effect of WL HpoA and SRHA. Possibly, these differences may relate to differences in experimental design (e.g., washed cell or growth cycle methylation) and/or the different bacteria used, but further tests are needed to explain this observation. We note that although our results were broadly consistent with those from previously published studies of different SRB strains, our longer incubation period of ~30 days may also preclude a direct comparison.

Here, the total amount of MeHg produced as a function of % NOM aromaticity (for any length of equilibration time in the case of F1 HpoA) was fit with a quadratic model (*r*^2^ = 0.887 and 0.999 for tracer and ambient Hg methylation, respectively), supporting the idea that MeHg production is variably but significantly correlated with the degree of aromatic functionality in each NOM isolate. The aromatic component of NOM has been shown to enhance the dissolution of cinnabar (Waples et al., 2005), affect the growth and stability of metal sulfide nanoparticulates in aquatic environments (Deonarine et al., 2011; Gerbig et al., 2011), and promote bioavailability of Hg for methylating microbes (Hall et al., 2008; Mitchell and Gilmour, 2008; Graham et al., 2012). We interpret the bioavailability of NOM-associated Hg for methylation to depend in a non-linear fashion with degree of hydrophobicity, apparently attaining a maximum at around 25–30% aromatic content. No other NOM compositional parameter could be as closely fit to the maximum Hg methylation data as aromaticity in this study.

Other studies have proposed an important role for dissolved hydrophobic HgS species, ${\text{HgS}}_{\left(\text{aq}\right)}^{0}$, in the bioavailability of Hg for methylation (e.g., Benoit et al., 1998, 2001b; Miller et al., 2007). While our results can neither confirm nor refute the presence of such species, they do indicate a close (but potentially complex) relationship between MeHg production and the degree of hydrophobicity in Hg-bearing NOM. Although NOM macromolecules are unlikely to be taken up by *D. propionicus* cells, recent work points to the possibility of the presence of HgS nanoparticles for uptake and potential methylation by microbial cells (Deonarine and Hsu-Kim, 2009; Gerbig et al., 2011; Graham et al., 2012, 2013; Zhang et al., 2012, 2014; Pham et al., 2014). NOM and small molecular weight organic acids have been shown to promote the suspension and stabilization of nanoparticulate mercury sulfide, with this nanophase HgS exhibiting a strongly hydrophobic functional nature (Deonarine and Hsu-Kim, 2009; Gondikas et al., 2010; Gerbig et al., 2011). These nanoparticles have been shown to form in association with NOM in the presence of ~3 μM sulfide (compared to 5–10 μM used in this experiment), although at between 3 and 5 orders of magnitude higher Hg(II) concentrations (3–100 μM; Deonarine and Hsu-Kim, 2009; Pham et al., 2014) than the environmentally typical levels used in this experiment (~1.5 nM). We therefore suggest that NOM-associated HgS nanoparticles could have been present as the bioavailable phase of Hg(II) for methylation in this experiment, although further study over a range of environmentally relevant Hg and sulfide concentrations, and using different bacteria, could be pursued. At any rate, nanoparticulate HgS may have to be small enough to enter the *D. propionicus* 1pr3 cell for intracellular methylation. Deonarine and Hsu-Kim (2009) and Pham et al. (2014) showed that nano-HgS stabilized by NOM could originate as 3–5 nm diameter precipitates (i.e., prior to aggregation), similar to the size of quantum dots known to be capable of entering bacterial cells (Kloepfer et al., 2005). Alternately, we speculate that Hg(II) may become disassociated from nano-HgS on or near cell surfaces, and then actively or passively transported across the cell wall.

As a note, while ~100 ng L^−1^ (final concentration) of tracer Hg (^200^Hg) was added to each bottle for each experimental condition, our analyses in some cases showed considerably less total dissolved Hg(II) was recovered, after filtration through 0.22 μm nylon syringe filters. The observed percentages of MeHg/filtered Hg_(*T*)_ were within the range of previously published values; however, we acknowledge the omission of mass balance calculations to be a limitation of this study. Although no mineral precipitation was observed, we acknowledge that some ^200^Hg(II) could have precipitated as colloidal or nanoparticulate HgS that remained suspended, possibly in association with cells and/or NOM isolates (Benoit et al., 2001a), and as aggregates >0.2 μm in diameter. Such a process would be consistent with the observations of other studies that showed the stabilization and aggregation of HgS nanoparticles and colloids in association with NOM (Ravichandran et al., 1999; Deonarine and Hsu-Kim, 2009; Slowey, 2010; Gerbig et al., 2011; Zhang et al., 2012). Recent work has shown that nanoparticulate HgS is more bioavailable for methylation than microparticulate HgS; however, over time nano-HgS precipitation and dissolution results in a mix of dissolved, nano-, and microparticulate forms of HgS that decrease its bioavailability (Zhang et al., 2012, 2014; Pham et al., 2014).

In the context of recently increased awareness of the effects of NOM and HS^−^ on the speciation of Hg(II) and other metals (cf. Aiken et al., 2011), our study points toward a potentially important role for NOM-associated nanoparticulate HgS in microbial Hg methylation and the biogeochemical mercury cycle. Recent studies using both microbial isolates and microcosm experiments have shown total HgS bioavailability decreasing with age as dissolved and nanoparticulate HgS partition to bulk-scale mineral particles and colloids (Zhang et al., 2012, 2014; Pham et al., 2014). Although nanoparticulate HgS species may be an important source of Hg for methylating microbes (Graham et al., 2012; Zhang et al., 2012, 2014; Pham et al., 2014), methylation rates may be influenced by the adsorption, complexation, and aggregation of HgS particulates by NOM (Aiken et al., 2011; Jonsson et al., 2012).

Interestingly, sites that attract NOM-Hg-sulfide aqueous or nanoparticulate species may also be present on bacterial cell surfaces or extracellular polymeric substances (EPS) (Benoit et al., 2001a). Anandkumar et al. (2012) used Fourier Transform Infrared Spectroscopy (FTIR) to support the interpretation that *D. propionicus* cells are capable of producing EPS that exhibited spectral features consistent with both carboxylate and thiol functional groups which can bind Hg(II). Certainly, several recent studies have shown that Hg(II) readily adsorbs to both live and dead cells, as well as EPS (e.g., François et al., 2012; Dash and Das, 2015). Furthermore, research has shown that metals and hydrophobic compounds can be partitioned separately between cell surfaces and biofilm (EPS) matrix (Flemming and Wingender, 2010), of which the latter may contain hydrophilic, hydrophobic and amphoteric sites (e.g., Sheng et al., 2010). Thus, the exact role of potential cell surface or EPS interactions with various Hg species, as well as cell growth phase and morphology (Moberly et al., 2012) remains to be investigated in detail with respect to mercury bioavailability for methylation. Mercury bioavailability may also depend upon the state of Hg redox cycling catalyzed by NOM functional groups (Zheng et al., 2012), potentially occurring simultaneously with sorption or hydrophobic interactions.

In summary, our work has shown that the degree of NOM hydrophobicity (mainly imparted from aromatic functional groups) strongly influenced the uptake and/or methylation of newly added (“tracer”) and aged/background (“ambient”) mercury. The observed variability in methylation efficiency probably reflects second-order and presumably kinetic effects on cellular Hg(II) uptake, possibly resulting from variability in the conformation state of the NOM macromolecules to which Hg was partitioned, or from the form of aged Hg-S complexes within the NOM. Other factors such as the relative sizes of new and ambient bioavailable Hg pools may also have influenced methylation efficiency. We note that our results can be considered usefully in the context of *in situ* environmental studies that have shown relatively enhanced reactivity with respect to methylation, although potentially lower aqueous mobility initially, for newly deposited Hg(II), relative to older “ambient” Hg(II) in soils and lake waters (e.g., Hintelmann et al., 2002; Chadwick et al., 2013; Oswald et al., 2014). A strong association with NOM in the conversion of reactive Hg(II) to MeHg is common to both laboratory-based and natural environment studies. Further experiments are necessary to elucidate the nature of Hg associations with NOM in Hg-NOM-HS^−^ systems and the effect of variable NOM composition and Hg-NOM-S aging on Hg methylation. Our work confirms a key role for NOM degree of hydrophobicity in controlling the bioavailability of either newly added or aged Hg(II) for uptake and methylation by a model sulfate-reducing bacterium.

## Author contributions

JM designed and conducted the experiment with supervision from ER and DK. GA contributed and advised on the NOM isolates. JM and JO conducted Hg and MeHg analyses with supervision from JD and DK. JM wrote the paper with contributions from CG, DK, GA, and ER.

### Conflict of interest statement

The authors declare that the research was conducted in the absence of any commercial or financial relationships that could be construed as a potential conflict of interest.
